# Real-World Evidence for the Safety and Efficacy of CGRP Monoclonal Antibody Therapy Added to OnabotulinumtoxinA Treatment for Migraine Prevention in Adult Patients With Chronic Migraine

**DOI:** 10.3389/fneur.2021.788159

**Published:** 2022-01-06

**Authors:** Laszlo Mechtler, Nicolas Saikali, Jennifer McVige, Olivia Hughes, Alexandra Traut, Aubrey Manack Adams

**Affiliations:** ^1^DENT Neurologic Institute, Amherst, NY, United States; ^2^ICON plc, Boston, MA, United States; ^3^Allergan, an AbbVie Company, Irvine, CA, United States

**Keywords:** calcitonin gene-related peptide, combination treatment, headache, onabotulinumtoxinA, migraine, prevention, safety

## Abstract

**Background:** OnabotulinumtoxinA and calcitonin gene-related peptide (CGRP) monoclonal antibodies (mAbs) target different migraine pathways, therefore, combination treatment may provide additional effectiveness for the preventive treatment of chronic migraine (CM) than either treatment alone. The objective of this study was to collect real-world data to improve the understanding of the safety, tolerability, and effectiveness of adding a CGRP mAb to onabotulinumtoxinA treatment for the preventive treatment of CM.

**Methods:** This was a retrospective, longitudinal study conducted using data extracted from a single clinical site's electronic medical records (EMR) of adult patients (≥18 years) with CM treated with ≥2 consecutive cycles of onabotulinumtoxinA before ≥1 month of continuous onabotulinumtoxinA and CGRP mAb (erenumab, fremanezumab, or galcanezumab) combination treatment. Safety was evaluated by the rate of adverse events (AE) and serious adverse events (SAE). The proportion of patients who discontinued either onabotulinumtoxinA, a CGRP mAb, or combination treatment, and the reason for discontinuation, if available, was collected. The effectiveness of combination preventive treatment was assessed by the reduction in monthly headache days (MHD). Outcome data were extracted from EMR at the first CGRP mAb prescription (index) and up to four assessments at ~3, 6, 9, and 12 months post-index. The final analyses were based on measures consistently reported in the EMR.

**Results:** EMR were collected for 192 patients, of which 148 met eligibility criteria and were included for analysis. Erenumab was prescribed to 56.7% of patients, fremanezumab to 42.6%, and galcanezumab to 0.7%. Mean (standard deviation [SD]) MHD were 20.4 (6.6) prior to onabotulinumtoxinA treatment and 14.0 (6.9) prior to the addition of a CGRP mAb (baseline). After real-world addition of a CGRP mAb, there were significant reductions in MHD at the first assessment (~3 months) (mean −2.6 days/month, 95% CI −3.7, −1.4) and at all subsequent visits. After ~12 months of continuous combination treatment, MHD were reduced by 4.6 days/month (95% CI −6.7, −2.5) and 34.9% of patients achieved ≥50% MHD reduction from index. AEs were reported by 18 patients (12.2%), with the most common being constipation (*n* = 8, 5.4% [onabotulinumtoxinA plus erenumab only]) and injection site reactions (*n* = 5, 3.4%). No SAEs were reported. Overall, 90 patients (60.8%) discontinued one or both treatments. The most common reason for discontinuing either treatment was lack of insurance coverage (40%); few (~14%) patients discontinued a CGRP mAb and none discontinued onabotulinumtoxinA due to safety/tolerability.

**Conclusion:** In this real-world study, onabotulinumtoxinA was effective at reducing MHD and the addition of a CGRP mAb was safe, well-tolerated and associated with incremental and clinically meaningful reductions in MHD for those who stayed on the combination treatment. No new safety signals were identified. Of those who discontinued, the majority reported lack of insurance coverage as a reason. Prospective real-world and controlled trials are needed to further evaluate the safety and potential benefits of this combination treatment paradigm for people with CM.

## Introduction

Chronic migraine (CM) is a complex, neurological disease impacting ~1–2% of the global population (1). The International Classification of Headache Disorders, 3rd edition (ICHD-3) defines CM as ≥15 monthly headache days (MHD) for >3 months over a 12 month period, with at least 8 headaches per month fulfilling the criteria for migraine with or without aura (2). Individuals with CM experience frequent debilitating migraine attacks, which prevent them from performing daily activities and significantly impact their quality of life. As a result, CM is associated with substantial societal and familial burdens (3, 4); and CM is associated with significant direct and indirect costs, leading to an economic burden for patients and healthcare systems (5–7).

The successful management of CM requires a holistic approach, which includes lifestyle modifications, trigger management, appropriate acute and preventive pharmacological treatments, and management of comorbidities (8–11). The goals of preventive treatment for CM include reducing the frequency, severity, duration, and disability of a migraine attack. Additionally, preventive treatment may alleviate some of the burdens associated with CM by improving function, reducing disability, reducing overall costs associated with migraine treatment, improving health-related quality of life, and reducing headache-related distress and psychological symptoms (10, 12). OnabotulinumtoxinA was first approved as a preventive treatment for CM in 2010 and since then, a robust body of evidence from clinical and real-world studies has demonstrated its safety, tolerability, and efficacy for CM prevention (13–17). Direct inhibition of CGRP pathways has emerged as a new target of both acute and preventive migraine treatments (18). In 2018, three subcutaneously injected monoclonal antibodies (mAbs) directed against CGRP or its receptor were approved by the US Food and Drug Administration (FDA) for migraine prevention. Erenumab (19) targets the CGRP receptor, while fremanezumab (20) and galcanezumab (21) directly target CGRP, preventing ligand binding to the CGRP receptor. In 2020, the FDA also approved eptinezumab, an intravenously administered mAb that also binds to the CGRP ligand (22, 23).

The pathophysiology of CM is complex, involving multiple pathways and receptors. As a result, though patients with CM often benefit from preventive treatment with onabotulinumtoxinA, they may continue to experience migraine attacks frequently enough to meet the criteria for receiving additional preventive treatments. The AHS recently published an updated position statement emphasizing the importance of developing preventive treatment plans to meet the individual needs of patients with migraine, which may involve combining older and newer treatments as well as complex and non-traditional approaches (11). The position statement reports that combination treatment with onabotulinumtoxinA and a CGRP mAb is probably effective, and CGRP therapy may be added to one or more established treatments based on clinical judgment provided the risk of drug-antibody interactions is considered minimal or non-existent (10, 11). Preclinical and clinical data suggest that combination treatment with onabotulinumtoxinA and a CGRP mAb could be additive or synergistic in the prevention of migraine due to the distinct mechanisms of action of the treatments (24–30).

To date, no randomized controlled clinical trials have evaluated the safety and efficacy of combination treatment with onabotulinumtoxinA and a CGRP mAb for migraine prevention. The objective of this study was to improve the understanding of the safety, tolerability, and potential benefits of adding a CGRP mAb to onabotulinumtoxinA as a combination preventive treatment regimen in adults with CM. To accomplish this, we collected real-world data from the electronic medical records (EMR) of patients treated with up to 12 months of continuous combination treatment at a single clinical site. Treatment benefits were based on effectiveness assessments available in patients' charts that are widely used and recognized as being reliable, accurate, and relevant to migraine.

## Materials and Methods

### Study Design

**Figure 1:**
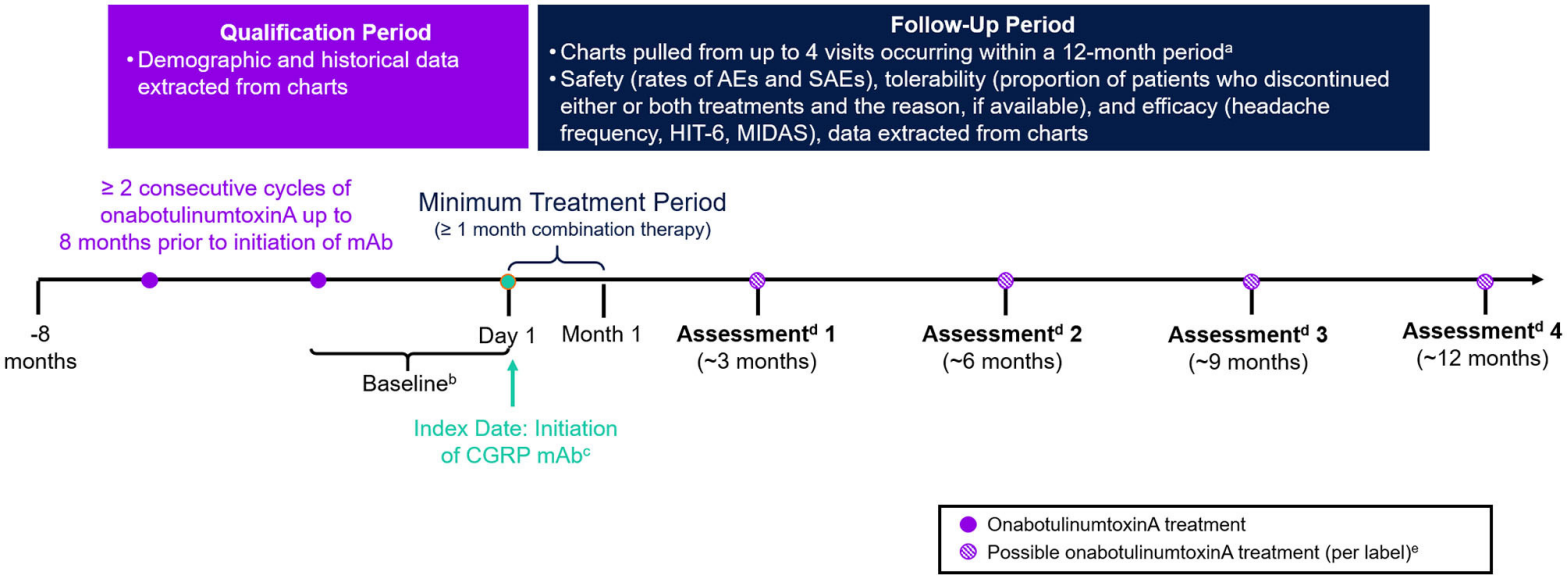
Study design. CGRP, calcitonin gene–related peptide; HIT-6, Headache Impact Test; mAb, monoclonal antibody; MIDAS, Migraine Disability Assessment. ^a^Not all patients had 4 visits or 12 months of data. ^b^Baseline assessments for outcome measures (e.g., headache day frequency, headache intensity, and disability) were collected from the visit at which the CGRP mAb was prescribed and reflect patient assessments during ~1–3 months prior to initiation of the CGRP mAb. ^c^CGRP mAbs were self-administered by subcutaneous injection. Per label, erenumab, fremanezumab, and galcanezumab are administered once monthly. ^d^Each assessment was based on data from chart extraction. ^e^OnabotulinumtoxinA treatment is not always administered per label.

This was a retrospective, non-interventional, longitudinal study conducted using data extracted from EMR of eligible patients treated at the DENT Headache Center (Buffalo, New York, USA) between June 1, 2018 and March 15, 2020. The center is part of the DENT Neurologic Institute, a high volume, private outpatient neurology group with a dedicated headache center involving board-certified, headache-medicine specialists. A schematic diagram of the study design is presented in Figure 1. The index date was defined as the start of combination treatment with onabotulinumtoxinA and a CGRP mAb and occurred between June 1, 2018 and March 15, 2019. A baseline period of 1–3 months prior to index was used to assess the effectiveness of onabotulinumtoxinA treatment monotherapy. Charts were reviewed up to 8 months prior to the index date to confirm eligibility. Patients were followed from the index date up to ~12 months post-index (corresponding to up to four treatments of onabotulinumtoxinA).

### Study Population

Eligible patients were identified based on a physician diagnosis of CM and met the following criteria: ≥18 years of age, received ≥2 consecutive treatments with onabotulinumtoxinA prior to starting CGRP mAb therapy, and received at least 1 month (one cycle) of combination treatment with onabotulinumtoxinA and a CGRP mAb. No exclusion criteria were established prior to data extraction. The target sample size was up to ~300 patients, the expected number of eligible patients treated at the participating site.

### Compliance With Ethics Guidelines

The New England Independent Review Board (IRB) reviewed the study protocol prior to study initiation and determined the study as exempt from review. According to the New England IRB, this research met the requirements for a waiver of consent. This study was conducted in accordance with current applicable regulations, International Conference of Harmonization guidelines, and local legal requirements, and complies with the ethical principles of the World Medical Assembly.

### Data Collection

Data were extracted from the eClinicalWorks system of EMR data from the DENT Neurologic Institute. The DENT Neurologic Institute provided de-identified EMR data of targeted patients to SyTrue to create a dataset containing study variables of interest using a Natural Language Processing (NLP) algorithm. The DENT Neurologic Institute then populated an approved data collection spreadsheet from the dataset and provided this to ICON Clinical Research Ltd. for analysis. Baseline demographics, migraine-relevant clinical history, concomitant migraine medication(s) use, and duration of onabotulinumtoxinA prior to starting CGRP mAb treatment were collected as potential explanatory or confounding variables. Treatment patterns were measured by onabotulinumtoxinA treatment dose and the duration between treatment doses and by the type of CGRP mAb prescribed, dose, and any change of type, dose, or regimen.

The reporting of adverse events (AE) and serious adverse events (SAE) were used to assess the safety of combination treatment with onabotulinumtoxinA and a CGRP mAb in this study. AE were considered to be any unfavorable and/or unintended sign (including an abnormal laboratory finding), symptom, or disease temporarily associated with the use of a pharmaceutical product that may or may not have a causal relationship with the treatment under investigation. SAE were defined as any AE occurring at any dose that results in any of the following outcomes: death, a life-threatening AE, inpatient hospitalization, or prolongation of hospitalization, a persistent or significant disability/incapacity, or a congenital anomaly/birth defect. Tolerability of combination treatment with onabotulinumtoxinA and a CGRP mAb was assessed by discontinuation of onabotulinumtoxinA treatment, discontinued or changed CGRP mAb treatment, or concurrent discontinuation of both treatments and the reported reason for discontinuation being safety or tolerability, when available.

Outcome measures to evaluate the potential benefits of combination treatment with onabotulinumtoxinA and a CGRP mAb were assessed at ~3, 6, 9, and 12 months post-index. Headache frequency was captured from the EMR with a 30- or 90-day denominator and standardized to a 30-day denominator (monthly headache days) for the analyses. Patients for whom a denominator was not indicated were reported as having unknown headache frequency. The effect of combination treatment on quality of life and disability was assessed with the 6-Item Headache Impact Test (HIT-6) and Migraine Disability Assessment (MIDAS), respectively. The HIT-6 is a 6-item questionnaire that assesses the impact of headache on patients' lives across six domains in the past 4 weeks (31). MIDAS is a 7-item measure of headache-related disability that assesses the number of days that migraine prevented or limited activities in the past 3 months (32).

### Statistical Analysis

Descriptive statistics were used to present and summarize the results of this study. Continuous variables were described by the mean, standard deviation (SD), median, minimum and maximum values, and categorical variables were summarized by the number (n) and percentage (%) of participants. Ninety-five percent confidence intervals (95% CIs) were calculated for all change in treatment benefit outcomes from baseline. For continuous variables, CIs of the mean difference between each follow-up measure from baseline were based on the pair of measures. Confidence intervals for the difference in proportions at each follow-up assessment as compared to baseline were calculated via McNemar's test.

Study follow-up duration was calculated as the time between the recorded index date and the earliest of the following: the date of the last recorded onabotulinumtoxinA treatment visit or its proceeding follow-up visit (within the study period), the date of the earliest onabotulinumtoxinA discontinuation, or the earliest CGRP mAb discontinuation. Consecutive cycles of treatment were defined as administrations of onabotulinumtoxinA <24 weeks apart; a gap of ≥24 weeks between administration was classified as discontinuation. For CGRP mAbs, the date of administration was estimated to occur according to treatment guidelines, unless discontinuation of the CGRP mAb was recorded in the patient EMR data.

Baseline demographic and clinical characteristics were tabulated and presented for all patients; for those with multiple variables for a specific value during the baseline period, the value collected closest to the index date was used. The safety of adding a CGRP mAb to onabotulinumtoxinA treatment as a combination preventive treatment for CM was assessed by the rates of AE and SAE during up to 12 months of continuous exposure to the combination treatment. AE are cumulative from the initiation of combination treatment to inform the safety findings during the follow-up period. Subgroup analyses for AE were conducted by index CGRP mAb therapy using a modified intent-to-treat approach, such that each patient remained in the subgroup determined by index CGRP mAb regardless of any change in CGRP mAb type during follow-up. Information related to onabotulinumtoxinA and/or CGRP mAb preventive treatment discontinuation and reason(s) for discontinuation, if available, were summarized as the proportion of patients who discontinued. Information related to change in CGRP mAb brand, dose, or regimen during follow-up was also summarized.

Mean (SD) and median (range) MHD and the percentage of patients who achieved controlled CM status (<15) vs. not (≥15 headache days/month) were calculated for each follow-up assessment. The effectiveness of combination treatment with onabotulinumtoxinA and a CGRP mAb was evaluated by changes in treatment outcomes from baseline for the following: mean monthly headache days; the percentage of patients with <15 (vs. ≥15) headache days/month; the percentage of patients with any reduction in MHD from baseline; and the percentage of patients with ≥25, ≥50, and ≥75% reduction in MHD. Mean (SD) and median (range) were calculated for HIT-6 and MIDAS total scores at baseline and follow-up assessments. HIT-6 scores were only available for <30% of patients and MIDAS scores for <10% of patients throughout follow-up. Since paired HIT-6 and MIDAS scores from baseline and post-index assessments were only available for up to four patients, no further analyses are reported for these data.

The final analyses were based on measures that were consistently reported in patients' EMR. Results are based on available patient data, and missing data values were not imputed. The only exception was for dates recorded without the day of the month (i.e., MM/YYYY format vs. DD/MM/YYYY format) the middle of the month (i.e., 15th day) was assumed for the purpose of calculating the duration between study events of interest. All analyses were performed by ICON Clinical Research Ltd using SAS® version 9.4.

## Results

### Study Population

**Figure 2:**
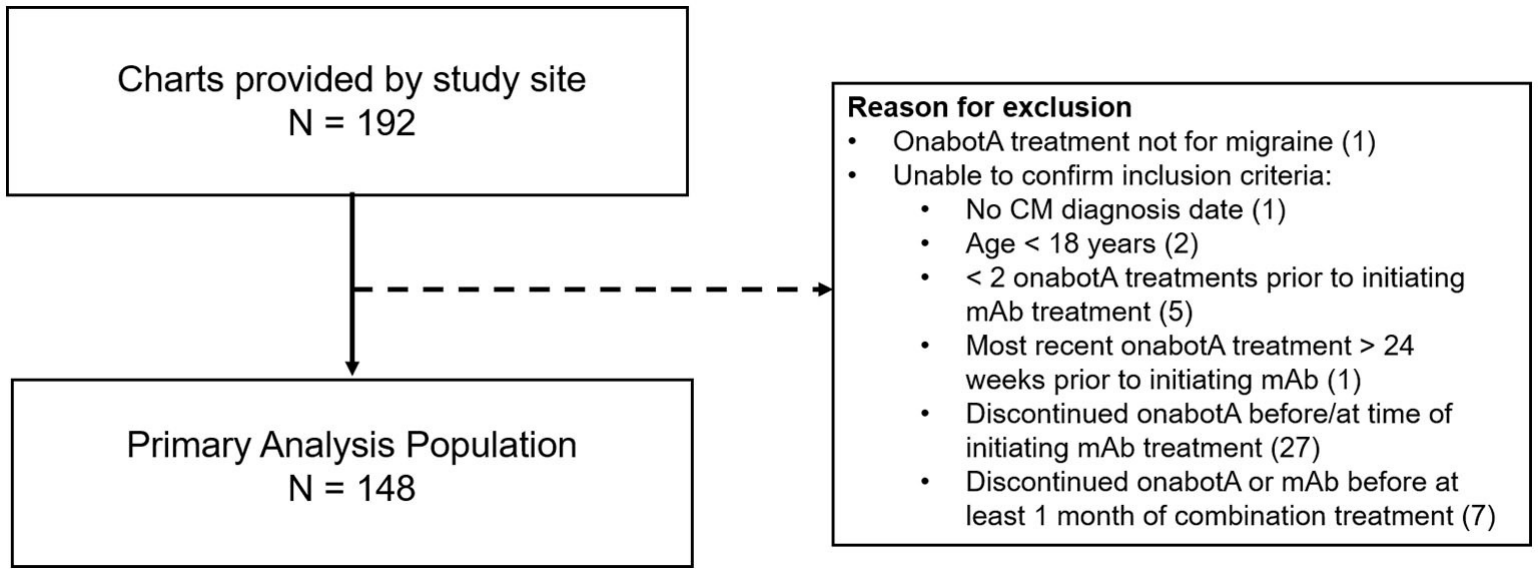
Primary analysis population. mAb, monoclonal antibody; onabotA, onabotulinumtoxinA. In the case of multiple inclusion criteria unconfirmed, patient is included in the first (*n*) only. This pertains to 2 patients.

Medical records were provided by the DENT Neurologic Institute for 192 patients deemed eligible for the study. As shown in Figure 2, 148 patient records met inclusion criteria and were used for analysis. Baseline demographic and clinical characteristics of the primary analysis population (*n* = 148), shown in Table 1, were consistent with those reported in the typical CM population. Patients were predominately female (93.2%) with a mean (SD) age of 46.9 (11.5) years. Patients initiated CGRP mAb combination treatment on average 2.6 years (range 0.2–9.1) after starting onabotulinumtoxinA treatment for CM. During the 3-month baseline period prior to initiating CGRP mAb therapy, the majority of patients were using concomitant migraine medications (*n* = 143/148, 96.6%) and had comorbid conditions (*n* = 142/148, 95.9%).

**Table 1:** Patient demographics and clinical characteristics prior to combination treatment.

**Parameter**	***N* = 148**
Age at study index (years), mean (SD)	46.9 (11.5)
Female, *n* (%)	138 (93.2)
Time since migraine diagnosis (years), mean (SD)	3.1 (2.1)
Time since onabotulinumtoxinA treatment initiation (years), mean (SD)	2.6 (2.0)
Time since most recent (pre-index) onabotulinumtoxinA injection (weeks)	5.0 (4.8)
**Index mAb treatment (mg)**, ***n*** **(%)**	
Erenumab (Aimovig), 140 mg	34 (23.0)
Erenumab (Aimovig), 70 mg	50 (33.8)
Fremanezumab (Ajovy), 225 mg	63 (42.6)
Galcanezumab (Emgality), 120 mg^a^	1 (0.7)
**Baseline concomitant medication use**, ***n*** **(%)**	143 (96.6)
***Acute*** **(top 3)**, ***n*** **(%)**	
Sumatriptan	41 (27.7)
Naproxen	27 (18.2)
Naratriptan	21 (14.2)
***Preventive*** **(top 3)**, ***n*** **(%)**	
Amitriptyline	25 (16.9)
Gabapentin	21 (14.2)
Topiramate	17 (11.5)
Comorbid conditions (any), *n* (%)	142 (95.9)

*mg, milligram; n, number; SD, standard deviation.*

*^a^Galcanezumab is administered as a 240 mg loading dose, followed by monthly doses of 120 mg*.

### Combination Treatment Characteristics

At index, the most commonly prescribed CGRP mAb was erenumab (*n* = 84/148 patients, 56.7%). Only one patient was prescribed galcanezumab at index (*n* = 1/148, 0.7%), the rest were prescribed fremanezumab (*n* = 63/148, 42.6%). Table 2 displays the average onabotulinumtoxinA dose administered at each post-index treatment visit, and the corresponding follow-up days from the previous treatment visit. OnabotulinumtoxinA treatment dose ranged from 120 to 200 units throughout follow-up, with a mean dose ranging from 165.3 to 167.0 units. Patients generally received onabotulinumtoxinA treatment at 3-month intervals, though with considerable variation across the study population.

**Table 2:** OnabotulinumtoxinA dosage during baseline and combination treatment.

	**Baseline** **(*N* = 148)**	**1st onabotA injection** **post-index** **(*N* = 127)**	**2nd onabotA injection** **visit post-index** **(*N* = 96)**	**3rd onabotA injection** **visit post-index** **(*N* = 73)**	**4th onabotA injection** **visit post-index** **(*N* = 45)**
**Follow up (days)** ^**a**^					
Mean (SD)	-	61.4 (29.0)	89.3 (9.3)	92.3 (16.6)	91.0 (17.6)
Median (range)	-	62 (7–155)	86 (61–121)	87.5 (40–168)	84.5 (60–168)
**OnabotulinumtoxinA dose (U)**					
Mean (SD)	166.6 (16.7)	166.5 (16.8)	165.3 (17.3)	167.0 (17.0)	166.1 (15.8)
Median (range)	155 (125–200)	155 (125–200)	155 (120–200)	165 (125–200)	165 (125–200)
Unknown				2 (2.7)	

*onabotA, onabotulinumtoxinA; SD, standard deviation; U, units.*

*^a^Follow up for 1st onabotA injection visit is calculated from index date; follow up for 2nd, 3rd, and 4th onabotA injection visit is calculated from 1st, 2nd and 3rd onabotA injection visits, respectively*.

### Effectiveness

Headache characteristics prior to onabotulinumtoxinA treatment and before the initiation of combination therapy with the addition of a CGRP mAb are shown in Table 3. Prior to onabotulinumtoxinA treatment, mean (SD) MHD were 20.4 (6.6) and 82 patients (55.4%) had ≥20 MHD. Treatment with onabotulinumtoxinA alone resulted in a statistically significant reduction in mean MHD of 6.4 days (95% CI 5.0–7.7). Over 88% of patients had any reduction in MHD from prior to onabotulinumtoxinA to index date, and 35.0% of patients (95% CI 27.2–43.4) had a 50% reduction in MHD with onabotulinumtoxinA treatment alone.

**Table 3:** Headache characteristics and disability at baseline.

**Characteristic**	**Primary analysis (*****N*** **=** **148)**	
	**Before onabotulinumtoxinA**	**Baseline (before mAb)**
**Headache frequency (days/month)**		
Mean (SD)	20.4 (6.6)	14.0 (6.9)
Median (range)	20 (2–30)	12 (0–30)
Change from pre-onabotA, mean (95% CI)		−6.4 (−7.8, −5.1)
**Headache days/month, n (%)**		
≤ 5	3 (2)	10 (6.8)
6–10	4 (2.7)	44 (29.5)
11–14	2 (1.4)	28 (18.2)
15–19	56 (37.8)	29 (19.6)
≥ 20	82 (55.4)	34 (23)
Unknown	1 (0.7)	4 (2.7)

*mAb, monoclonal antibody; MHD, monthly headache day; OnabotA, onabotulinumtoxinA; SD, standard deviation*.

**Figure 3:**
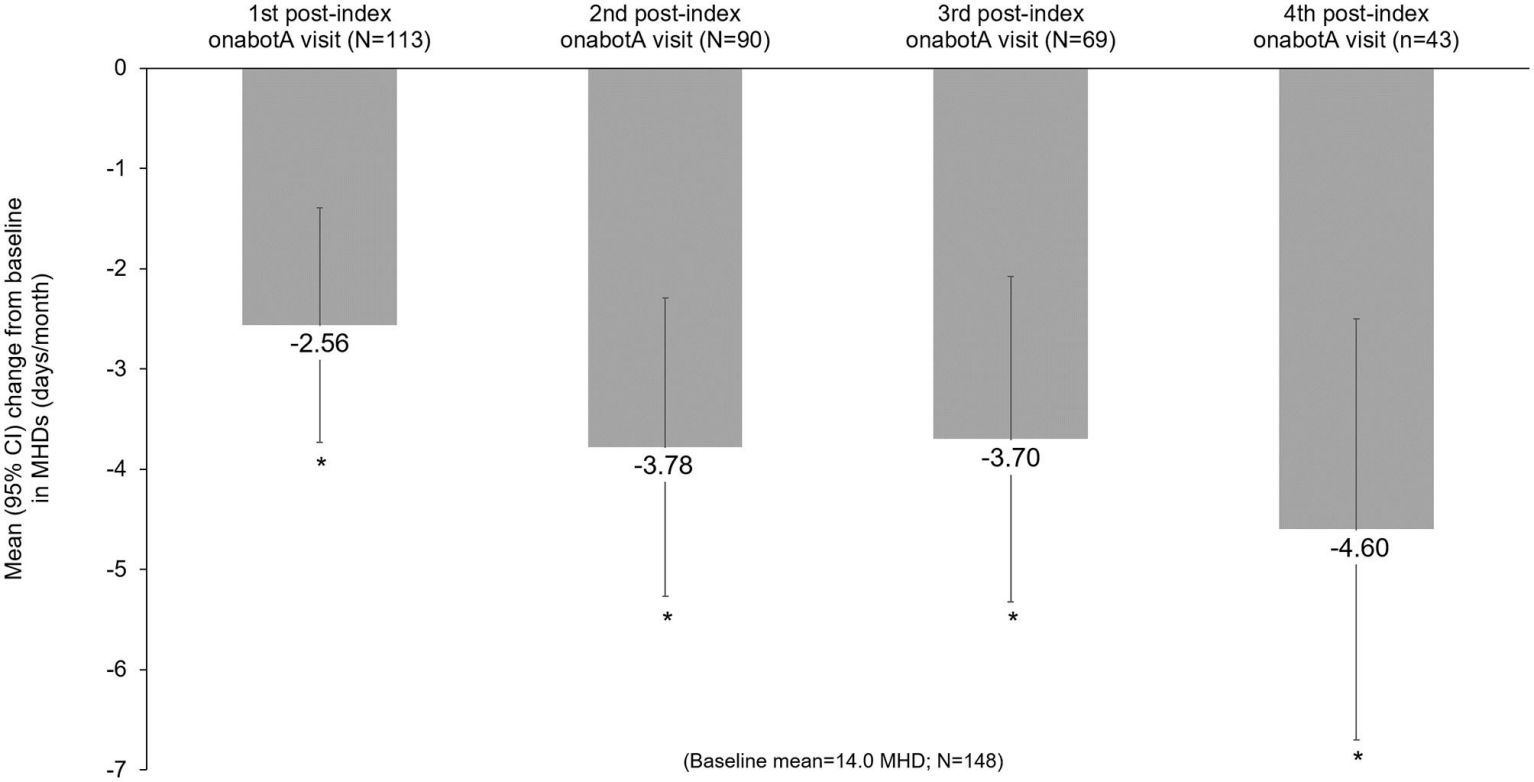
Mean change from baseline in monthly headache frequency during combination treatment with onabotulinumtoxinA and a CGRP mAb. CGRP, calcitonin gene-related peptide; CI, confidence interval; CM, chronic migraine; mAb, monoclonal antibody; MHD, monthly headache days; onabotA, onabotulinumtoxinA. *Indicates statistical significance (i.e., 95% CI does not include zero).

**Figure 4:**
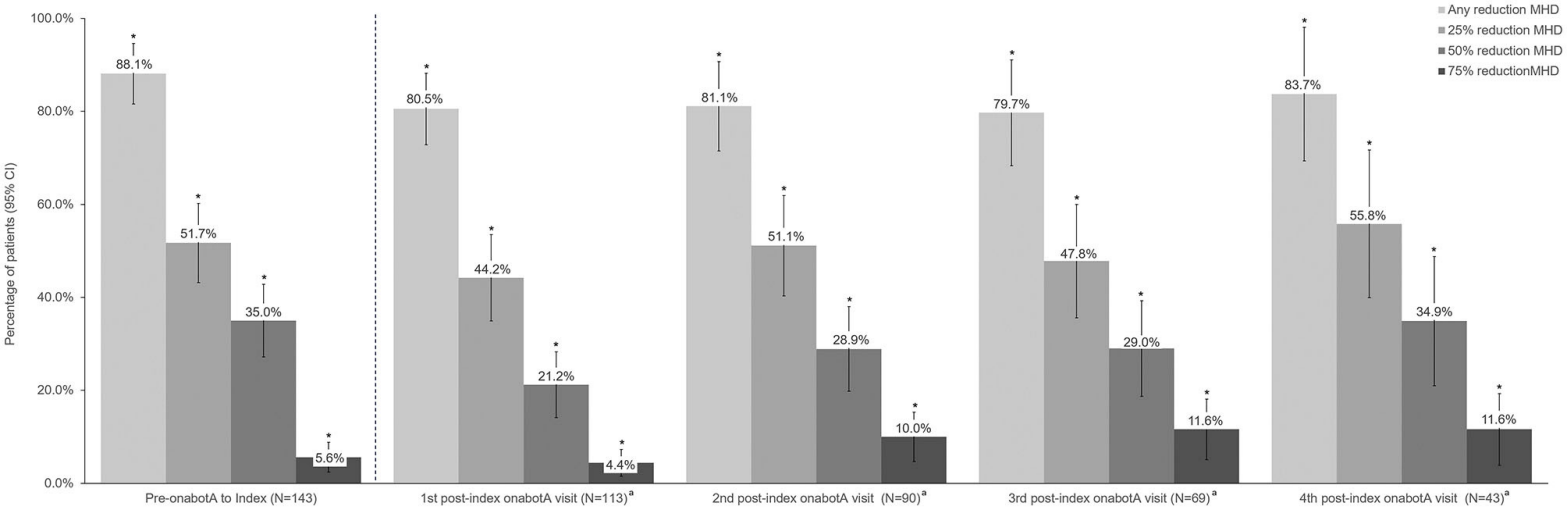
Percent reduction in monthly headache frequency prior to onabotulinumtoxinA treatment and during 12 months of combination treatment with onabotulinumtoxinA and a CGRP mAb. CGRP, calcitonin gene-related peptide; CI, confidence interval; MHD, monthly headache days; onabotA, onabotulinumtoxinA. ^a^Difference from baseline. *Indicates statistical significance (i.e., 95% CI do not include zero).

**Figure 5:**
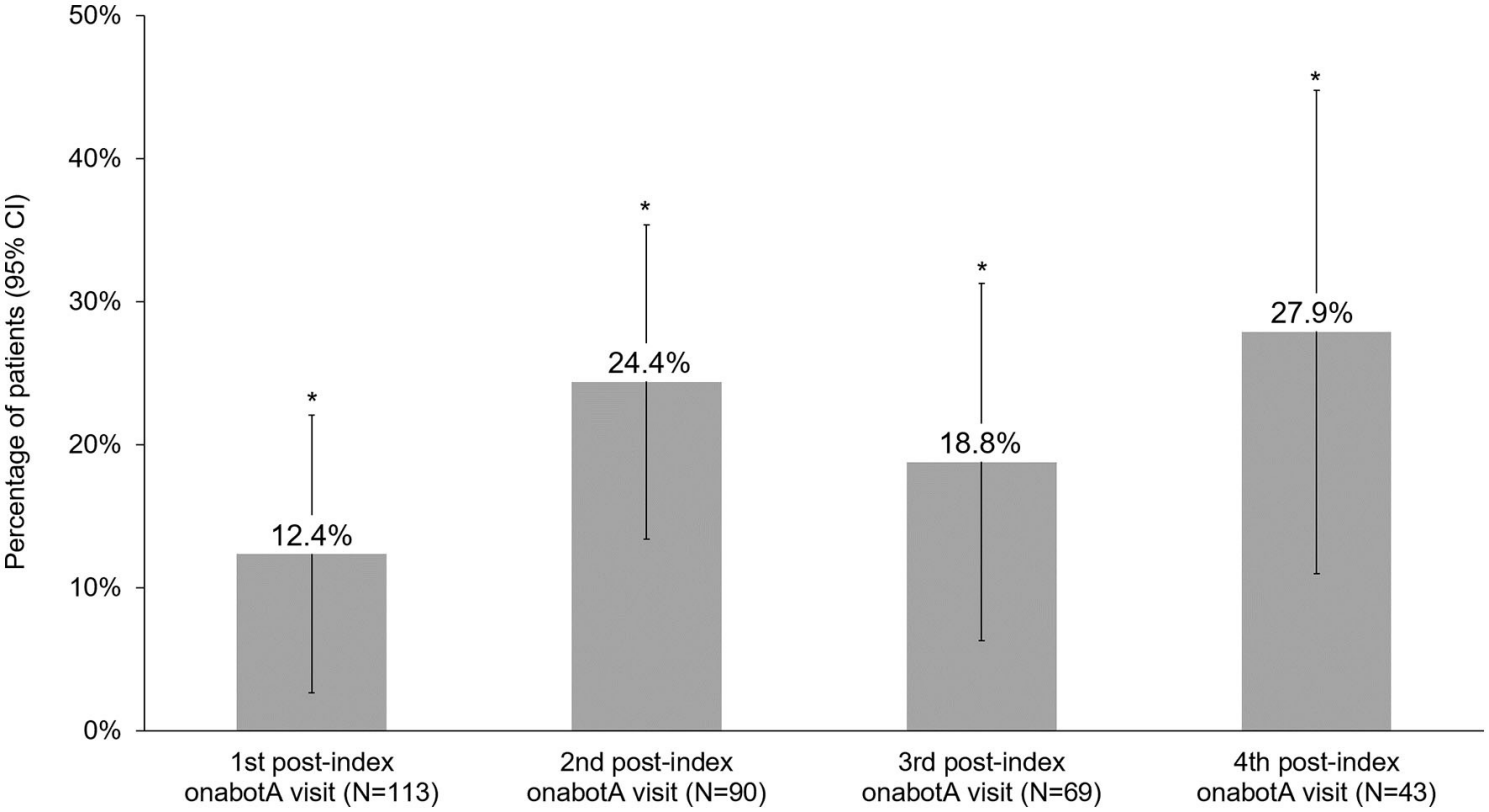
Proportion of patients who achieved controlled chronic migraine (<15 headache days/month) status on combination treatment with onabotulinumtoxinA and CGRP mAb. CGRP, calcitonin gene-related peptide; CI, confidence interval; mAb, monoclonal antibody; onabotA, onabotulinumtoxinA. *Indicates statistical significance (i.e., 95% CI does not include zero).

Headache frequency data collected at pre-CGRP mAb baseline and four post-index assessments over ~12 months are presented in Table 4. The addition of a CGRP mAb to onabotulinumtoxinA treatment for CM resulted in incremental reductions in MHD at all assessments, with a mean (SD) of 14 (6.9) MHD at baseline to 10.0 (6.5) at the 4th post-index assessment. Overall, there was a statistically significant reduction in MHD from pre-CGRP mAb baseline to each post-index assessment during follow-up (Figure 3). After ~12 months of combination treatment, MHD decreased by a mean of 4.6 days (95% CI 2.5–6.7) in patients with headache frequency available at that time point. At the 4th assessment (~12 months), 83.7% of patients (95% CI 69.3–93.2) achieved any reduction in MHD and 34.9% (95% CI 21.0–50.9) achieved ≥50% reduction in MHD from baseline with combination treatment for CM (Figure 4). As shown in Figure 5, the proportion of patients who achieved controlled CM status (<15 MHD) increased significantly from baseline at each consecutive post-index assessment. After ~12 months of combination treatment, the proportion of patients with <15 MHD was 27.9% higher than baseline.

**Table 4:** Headache frequency during baseline and combination treatment.

	**Baseline** **(*N* = 148)**	**1st post-index** **assessment** **(*N* = 127)**	**2nd post-index** **assessment** **(*N* = 96)**	**3rd post-index** **assessment** **(*N* = 73)**	**4th post-index** **assessment** **(*N* = 45)**
**Follow up (days)** ^**a**^					
Mean (SD)	-	61.4 (29.0)	89.3 (9.3)	92.2 (16.7)	90.9 (17.8)
Median (range)	-	62 (7–155)	86 (61–121)	87 (40–168)	84 (60–168)
Patients with frequency data, n (%)	144 (97.3)	115 (90.6)	90 (93.8)	69 (94.5)	43 (95.6)
**Headache frequency (days/month)**					
Mean (SD)	14.0 (6.9)	11.6 (6.3)	10.4 (6.7)	10.1 (6.4)	10.0 (6.5)
Median (range)	12 (0–30)	10 (1–30)	9 (2–30)	10 (1–30)	8 (1–30)
Unknown, n (%)	4 (2.7)	12 (9.4)	6 (6.3)	4 (5.5)	2 (4.4)
**Headache frequency categories, n (%)** ^**b**^					
≥15 HA days/month	63 (43.8)	36 (31.3)	20 (22.2)	16 (23.2)	8 (18.6)
<15 HA days/month	81 (56.3)	79 (68.7)	70 (77.8)	53 (76.8)	35 (81.4)

*HA, headache; onabotA, onabotulinumtoxinA; SD, standard deviation.*

*^a^Follow up for 1st onabotA injection visit is calculated from index date; follow up for 2nd, 3rd, and 4th onabotA injection visit is calculated from 1st, 2nd, and 3rd onabotA injection visits, respectively.*

*^b^Denominator is patients with frequency data*.

### Safety

Safety data reported over the entire follow-up duration is presented for the total analysis population and stratified by index CGRP mAb type in Table 5. Throughout a mean follow-up period of 207 days (range 0–485 days), 18 patients (12.2%) experienced at least one AE. Two patients (1.4%) who received erenumab at index experienced 2 AE and 1 patient (0.7%) who received fremanezumab at index experienced 4 AE. The most prevalent AE was constipation, experienced by 5.4% of all patients, all of whom received erenumab at index. The second most common AE was an injection site reaction, experienced by 3.4% of all patients.

**Table 5:** Adverse events for the overall study population and stratified by index CGRP mAb type.

	**Total**	**Erenumab**	**Galcanezumab**	**Fremanezumab**
	**(*N* = 148)**	**(*N* = 84)**	**(*N* = 1)**	**(*N* = 63)**
**Follow up (days)**				
Mean (SD)	207.1 (121.1)	210.1 (121.7)	128 (n/a)	204.3 (121.6)
Median (range)	224.5 (0–485)	236.5 (0–393)	128 (128–128)	220 (0–485)
Adverse event present (yes), n (%)	18 (12.1)	12 (14.3)	0 (0)	6 (9.5)
**Number of AEs, n (%)**				
0	131 (87.8)	72 (85.7)	1 (100)	57 (90.5)
1	15 (10.1)	10 (11.9)		5 (7.9)
2	2 (1.4)	2 (2.4)		1 (1.6)
4	1 (0.7)			
**AEs, n (%)**				
Constipation	8 (5.4)	8 (9.4)		3 (4.8)
Injection site reaction^a^	5 (3.4)	2 (2.4)		2 (3.2)
Muscular weakness	2 (1.4)			1 (1.6)
Other^b^	2 (1.4)	2 (2.4)		1 (1.6)
Rash	2 (1.4)	1 (1.2)		1 (1.6)
Cramps, muscle spasms	1 (0.7)			1 (1.6)
Dizziness	1 (0.7)			
Injection site pain	1 (0.7)	1 (1.2)		
Hypertension	1 (0.7)			

*^a^Includes hives, redness, welt, burning, swelling, and/or itching at injection site.*

*^b^Other AEs include tingling/swollen lips, increase in headaches*.

### Treatment Discontinuation

Discontinuation of CGRP mAb and/or onabotulinumtoxinA treatment, as well as changes in CGRP mAb treatment during follow-up, are presented in Table 6. Overall, 90 patients (60.8%) discontinued onabotulinumtoxinA or a CGRP mAb, including one patient who discontinued both treatments simultaneously. More patients discontinued CGRP mAb than onabotulinumtoxinA treatment. Of those who discontinued either treatment, six discontinuations were reported at or immediately following the 4th follow-up assessment. Additionally, 19 patients were lost to follow-up. Forty-two patients (28.4%) discontinued onabotulinumtoxinA treatment. The primary reasons for discontinuation were lack of effect (38.1%) and lack of insurance coverage (28.6%). Fifty patients (33.8%) discontinued CGRP mAb treatment, with the most common reason reported being lack of insurance coverage (50.0%), followed by lack of efficacy (30.0%), and safety/tolerability (14.0%). Changes in CGRP mAb brand, dose, and regimen were not common in this patient population. Throughout follow-up, 19 patients (12.8%) changed CGRP mAb brand once, 11 patients (7.4%) changed CGRP mAb dose, and no patients changed CGRP mAb regimen.

**Table 6:** Combination treatment changes and discontinuation during follow-up.

	**Total post-index, per patient (*N* = 148)**
**Follow-up (days)**	
Mean (SD)	207.1 (121.1)
Median (range)	224.5 (0–485)
Any discontinuation (onabotA or mAb), n (%)	90 (60.8%)
Discontinuation of onabotA therapy, n (%)	42 (28.2%)
**Follow-up duration until discontinuation (days)**	
Mean (SD)	132.1 (110.2)
Median (range)	101 (0–340)
**Reason for discontinuation** ^**a**^ **, n (%)**	
Lack of effect	16 (38.1%)
Lack of insurance coverage	12 (28.6%)
Analysis derived^b^	12 (28.6%)
Other/unknown	2 (4.8%)
Discontinuation of mAb therapy, n (%)	50 (33.8%)
**Follow-up duration until discontinuation (days)**	
Mean (SD)	185.0 (96.9)
Median (range)	177.5 (44–392)
**Reason for discontinuation** ^**a**^ **, n (%)**	
Lack of effect	15 (30%)
Lack of insurance coverage	25 (50%)
Safety/tolerability	7 (14%)
Other/unknown	4 (8%)
Change in mAb brand^c^, n (%)	19 (12.8%)
Number of changes in mAb brand during follow-up, median (range)	1.0 (1–1)
Change of mAb dose^c^, n (%)	11 (7.4%)
Number of changes in mAb dose during follow-up, median (range)	1.0 (1–1)
**Change of mAb regimen, n (%)**	
No	148 (100%)

*^a^Multiple reasons for discontinuation were allowed and therefore may not sum to 100%.*

*^b^Patients were considered to discontinue onabotA if consecutive injections occurred >24 weeks apart.*

*^c^Indicates patient had at least one change in mAb brand/dose/regimen*.

## Discussion

Chronic migraine is a debilitating disease with a complex pathophysiology, involving multiple pathways and receptors. As a result, though patients with CM often benefit from preventive treatment with onabotulinumtoxinA, they may continue to experience migraine attacks frequently enough to meet the criteria for receiving additional preventive treatments. AHS recently published an updated position statement emphasizing the importance of developing preventive treatment plans to meet the individual needs of patients with migraine, which may involve combining older and newer treatments as well as complex and non-traditional approaches (11).

The addition of a CGRP mAb to onabotulinumtoxinA treatment for CM is routinely done in clinical practice among neurologist and headache specialists, however, data on the safety and efficacy of this treatment paradigm is limited. To date, no prospective clinical trials and only a limited number of real-world studies have examined this treatment approach (33–38). The AHS position statement reports that combination treatment with onabotulinumtoxinA and a CGRP mAb is probably effective, and CGRP therapy may be added to one or more established treatments based on clinical judgment provided the risk of drug-antibody interactions is considered minimal or non-existent (10, 11). Preclinical and clinical studies suggest that combination treatment with mechanistically distinct preventive treatments could have additive or synergistic effects in migraine prevention (26). Preclinical data show that onabotulinumtoxinA and CGRP mAbs prevent the activation of different types of pain fibers involved in migraine: onabotulinumtoxinA primarily prevents the activation of unmyelinated C-fibers (30) and CGRP mAbs mainly prevent the activation of thinly myelinated Aδ-fibers (29). Real-world studies have provided initial evidence of clinical benefit with the addition of a CGRP mAb to onabotulinumtoxinA treatment for CM (33–38).

In this retrospective, real-world study of 148 patients, the addition of a CGRP mAb (erenumab, galcanezumab, or fremanezumab) to onabotulinumtoxinA treatment as a combination preventive treatment for CM was generally well-tolerated and no new safety signals were identified. Patients discontinued CGRP mAb therapy more frequently than onabotulinumtoxinA (33 vs. 28%), though all patients included in this study received at least two onabotulinumtoxinA treatments before initiating combination treatment, which may partially explain this difference as patients not tolerating onabotulinumtoxinA were likely precluded from the study. The primary drivers of discontinuation of either treatment were lack of insurance coverage (40%) and lack of effect (34%). Only seven patients (14%) discontinued a CGRP mAb due to safety/tolerability and none discontinued onabotulinumtoxinA for this reason. Throughout follow up, only 12% of patients changed CGRP mAb brand and 7% changed CGRP mAb dose. While on combination preventive treatment, 12% of patients experienced one or more AE. The most common AE reported was constipation, experienced by 9.5% of patients prescribed erenumab at index. Injection site reactions were the next most common AE, experienced by 3.4% of total patients. No SAE were reported, and no new safety signals were identified.

The safety profile of combination treatment in this real-world study is consistent with that observed in prior analyses of each treatment alone and other real-world studies of CGRP mAbs added to onabotulinumtoxinA treatment for CM. In a similar study of combination preventive treatment of CM with onabotulinumtoxinA and a CGRP mAb by Blumenfeld et al. (36) 28% of patients experienced an AE; the only one reported by ≥5% of patients was constipation. Another retrospective chart review of patients prescribed a CGRP mAb while receiving onabotulinumtoxinA for CM by Cohen et al. (34) reported that AEs occurred in 8.5% of patients and included constipation, injection site reaction, and/or fatigue. The incidence of constipation was higher (34%) in a real-world observational study of 158 patients with CM treated with erenumab with or without other preventives or onabotulinumtoxinA (37). Constipation is a known adverse event associated with erenumab (19). Gastrointestinal and other AE are less common with the other CGRP mAbs prescribed in this study, occurring at rates similar to those observed with placebo in clinical trials (39–41). The most common AE reported with onabotulinumtoxinA for the treatment of CM in clinical trials were neck pain (9%) and headache (5%) (13, 14).

Clinically meaningful treatment benefits were observed with onabotulinumtoxinA prior to mAbs initiation and additional statistically significant benefits were observed after adding a CGRP mAb in patients who remained on combination treatment. Prior to the addition of a CGRP mAb, patients experienced a mean reduction of 6.4 MHD with onabotulinumtoxinA treatment alone. This mean is slightly lower than the average decrease in the PREEMPT trials, though it is important to note that mean baseline MHD were slightly higher in the PREEMPT trials than the present analysis (13, 14). Real-world CM patients are often more complicated than those enrolled in clinical trials due to the severity of disease and comorbidities. In particular, those identified for combination treatment may represent a severe and refractory subset of CM patients. Due to the nature and severity of CM, the patients in this study still experienced an average of 14 MHD at the initiation of combination treatment. The addition of a CGRP mAb to onabotulinumtoxinA treatment for CM resulted in significant and clinically meaningful reductions in MHD at each post-index assessment compared to baseline. Mean reductions of 3.8–4.6 days per month after 6–12 months of combination treatment were observed. After ~12 months of combination treatment, the proportion of patients who achieved controlled CM status (<15 MHD) was 27.9% higher than baseline, and over 30% of patients achieved at least a 50% reduction in MHD.

These findings are consistent with the results of other real-world studies evaluating the safety and efficacy of combination treatment with onabotulinumtoxinA and a CGRP mAb. The recent publication of a similar study by Blumenfeld et al. (36) showed a 3.5–4 day reduction in MHD over 6–12 months of combination treatment after an initial mean reduction from 21.5 MHD to 12.1 MHD with onabotulinumtoxinA alone. A retrospective analysis by Armanious et al. (33) recently demonstrated a 6.8–8.1 reduction in MHD in patients with CM who received 1–3 months of combination treatment with erenumab and onabotulinumtoxinA. The 78 patients included in this analysis had a mean (SD) of 22.5 (8.7) MHD while receiving onabotulinumtoxinA alone and likely represent a more refractory population, possibly explaining the greater reduction in MHD observed with combination treatment in this study. Boudreau (37) recently conducted an observational study comparing the effect of combination treatment with erenumab as an add-on therapy to onabotulinumtoxinA to the individual treatments alone. He found that the addition of erenumab to a preventive therapy was more effective to reduce MHD than erenumab alone. As in our study, patients in a retrospective review of medical records by Cohen et al. (34) experienced a significant (*p* < 0.001) reduction in MHD with onabotulinumtoxinA treatment and an additional reduction of 5.7 MHD (*p* < 0.001) with the addition of a CGP mAb.

In this study, lack of treatment insurance coverage was the most common reason for discontinuation of either onabotulinumtoxinA or a CGRP mAb. Insurance coverage for medication is a challenge pervasive throughout headache medicine and an issue that has been cited as a potential cause for increased use of emergency room care, infusion centers, and inpatient admissions (42), resulting in increased financial burden on patients and healthcare systems. Further, suboptimal migraine treatment can lead to increases in headache frequency and severity. Evidence suggests that ineffective migraine treatment is associated with chronification (43), leading to increased challenges longitudinally with treatment effectiveness and headache resolution. Unfortunately, patients with CM are often forced to choose by payers between two clinically-proven, beneficial treatment modalities, which data suggests are safe, additive, and possibly synergistic when combined. The patients described above undoubtedly represent the most severe cases of CM that overutilize direct medical resources and thereby, incur the highest costs. Examination by payers of the cost-benefit ratio of treatment options for these patients is warranted and may justify a more aggressive approach for the treatment of refractory CM. The data presented herein provide additional evidence that the combination use of onabotulinumtoxinA and a CGRP mAb for the preventive treatment of CM was generally well-tolerated, suggesting that the subset of patients with refractory CM could experience clinical benefit with such a combination regimen, without an increase in the side-effect profile.

Several limitations should be considered when interpreting the results of this study. First, data used for this real-world, retrospective study were collected for the purpose of health care, not research. As a result, variables of interest may have been missing as the result of several circumstances, including the data not being applicable for the specific patient, not available in the medical chart, or the data available in the medical chart but not captured by the data extraction processes. Additionally, these findings reflect the study site's clinical practice chart documentation, and some variables of interest, such as acute medication consumption, were not collected. A prospective study design that collects a daily patient diary could capture this information to assess acute treatment reduction and pharmacoeconomic considerations. Not all patients contributed data to all four post-index assessments due to loss to follow-up and discontinuation, which, though common and expected in real-world studies, can introduce bias. As a result, the data from later time points may reflect a responder population that does not capture the outcomes of patients who did not respond to treatment. In addition, study outcome measures were not consistently reported, and reporting methods may have varied across patients. Consistency of treatment with both onabotulinumtoxinA and CGRP mAbs may also have varied between patients. OnabotulinumtoxinA treatment may not always be administered per label, and some variation in dosage and timing of injections is likely. Patient compliance with CGRP mAb injections was not documented as this information is not routinely collected in the clinical setting. Therefore, the exact timing of the initiation of combination treatment is unknown and assessments may not accurately reflect 3, 6, 9, and 12 months of combination use. Lastly, patients in this study were prescribed a CGRP mAb during the 1st year following FDA approval; therefore, the distribution of CGRP mAb brands depended largely on the time of product release and the market availability of each product. For example, erenumab was the first FDA-approved treatment, which most patients (56.8%) received. Consequently, the number of patients treated with fremanezumab and galcanezumab was insufficient to allow for comparisons between different CGRP mAb brands.

## Conclusion

These real-world data demonstrate that the combination use of onabotulinumtoxinA and a CGRP mAb for the preventive treatment of CM was generally well-tolerated, with no new safety signals identified in patients who were able to stay on the combination. This combination treatment paradigm was associated with additional, clinically meaningful improvements in headache frequency in patients with chronic migraine compared to treatment with onabotulinumtoxinA alone. Discontinuation of one or both treatments was primarily driven by a lack of insurance coverage. Additional real-world studies and prospective controlled trials are needed to further assess the safety and additive or synergistic benefits of the addition of a CGRP mAb to onabotulinumtoxinA treatment for people with CM.

## Data Availability Statement

The raw data supporting the conclusions of this article will be made available by the authors, without undue reservation.

## Ethics Statement

The New England Independent Review Board reviewed the study protocol prior to study initiation and determined the study as exempt from review. Written informed consent for participation was not required for this study in accordance with the national legislation and the institutional requirements.

## Author Contributions

LM, NS, JM, and OH participated in data acquisition. All authors participated in the study conception, design, data analysis, interpretation, read, and approved the final manuscript.

## Funding

This research was supported by Allergan, an AbbVie Company.

## Conflict of Interest

LM has received personal compensation for consulting, serving on a scientific advisory board, speaking, research affiliation, or other activities with Alder Pharmaceuticals, Allergan (now AbbVie Inc.), Amgen, Avanir, Biohaven, Boston Biomedical Inc., CellDex, DelMar Pharmaceuticals, electroCore, Novartis, Orbis Pharmaceuticals, Promius, Teva Pharmaceuticals, and Jushi; he has financial interest in Jushi. NS has served as speaker and/or advisory board member for Allergan (now AbbVie Inc.), Amgen, Avanir, Biohaven, Currax, Depomed, Egalet, GammaCore, Eli Lilly, Lundbeck, Pernix, Promius, Supernus, and Teva. JM has served as a speaker and/or received research support from Allergan (now AbbVie Inc.), Amgen/Novartis, Avanir, Biohaven, Eli Lilly, Lundbeck, Theranica, Amneal and Teva. OH is an employee of ICON plc. AT and AA are full-time employees of AbbVie Inc., and may hold AbbVie stock. The authors declare that this study received funding from Allergan, an AbbVie Company. Employees of AbbVie participated in the research, interpretation of data, review of the manuscript, and the decision to submit for publication.

## Publisher's Note

All claims expressed in this article are solely those of the authors and do not necessarily represent those of their affiliated organizations, or those of the publisher, the editors and the reviewers. Any product that may be evaluated in this article, or claim that may be made by its manufacturer, is not guaranteed or endorsed by the publisher.
